# Meta-analytical insight on probiotic metabolites and inflammatory markers in diabetes

**DOI:** 10.3389/fcimb.2025.1677671

**Published:** 2025-09-22

**Authors:** Yanpeng Xie, Yingkang Zheng, Fan Jiang, Xiaojun Cai

**Affiliations:** ^1^ Department of Endocrinology, Heilongjiang Academy of Traditional Chinese Medicine, Harbin, Heilongjiang, China; ^2^ The First Clinical Medical College, Heilongjiang University of Chinese Medicine, Harbin, Heilongjiang, China; ^3^ General Office, Heilongjiang Provincial Administration of Traditional Chinese Medicine, Harbin, Heilongjiang, China

**Keywords:** systemic inflammation, diabetes mellitus, gut microbiota, short-chain fatty acids (SCFAs), inflammatory markers

## Abstract

**Introduction:**

Systemic inflammation is a hallmark of diabetes mellitus and contributes to insulin resistance and disease progression. Emerging evidence suggests that gut microbiota and their metabolites, particularly short-chain fatty acids (SCFAs), play a crucial role in modulating immune responses. Probiotics and synbiotics are increasingly explored for their potential to mitigate inflammation via microbiota-targeted mechanisms. This study aims to evaluate the effects of probiotic and synbiotic supplementation on inflammatory markers and microbial metabolites in individuals with type 1 and type 2 diabetes through meta-analytical techniques.

**Methods:**

A total of 46 randomized controlled trials (RCTs) comprising 3,580 diabetic patients were included following PRISMA guidelines. Meta-analyses were performed using random-effects models to assess changes in inflammatory markers (CRP, IL-6, TNF-α, IL-10) and SCFA levels (butyrate, propionate, acetate). Subgroup analyses and meta-regressions were conducted to identify effect modifiers such as intervention duration, formulation type (probiotic vs. synbiotic), and SCFA concentrations.

**Results:**

Probiotic/synbiotic interventions led to significant reductions in CRP (SMD = –0.54), IL-6 (SMD = –0.41), and TNF-α (SMD = –0.48), along with an increase in IL-10 (SMD = +0.38). SCFA levels rose significantly, with butyrate showing the strongest effect (SMD = +0.46). Meta-regression revealed that butyrate levels, synbiotic use, and intervention duration ≥8 weeks were strong predictors of anti-inflammatory efficacy. Multi-strain and synbiotic interventions were more effective than single-strain or probiotic-only formulations. Sensitivity analyses confirmed the robustness of findings, and publication bias was minimal.

**Discussion:**

These findings support the adjunctive use of targeted, SCFA-oriented probiotic formulations (e.g., *Lactobacillus plantarum, Lactobacillus casei, Bifidobacterium longum* with inulin/FOS, ≥10^9–10^10 CFU/day) to mitigate metabolic inflammation alongside standard care. Strain- and dose-standardized RCTs should confirm impacts on glycemic and cardiometabolic outcomes.

## 1 Introduction

Diabetes mellitus (DM) represents the pressing global health challenges, with rising incidence and prevalence rates posing a significant burden on healthcare systems (Arokiasamy et al., 2021). It is heterogeneous metabolic disorder characterized primarily by persistent hyperglycemia, which arises from defects in insulin secretion, insulin action, or combination of both. Type 1 diabetes (T1D) is predominantly autoimmune in origin, marked by destruction of pancreatic β-cells, while type 2 diabetes (T2D) is largely associated with insulin resistance and chronic metabolic stress (Eizirik et al., 2020). The two forms of diabetes have distinct etiologies, they converge in common pathogenic feature systemic, low-grade inflammation which contributes to both the onset and progression of the disease and its complications.

Inflammation in diabetes is driven by various cytokines and acute-phase reactants, including C-reactive protein (CRP), tumor necrosis factor-alpha (TNF-α), and interleukin-6 (IL-6), all of which serve as the biomarkers of immune activation (Bashir et al., 2020). These inflammatory mediators not only reflect disease severity but also actively participate in disrupting the insulin signaling pathways, impairing glucose uptake, and promoting endothelial dysfunction and atherosclerosis. Moreover, chronic inflammatory state in diabetic patients can exacerbate comorbidities in cardiovascular disease, nephropathy, and neuropathy, making it critical therapeutic target beyond glycemic control.

In recent years, a growing body of research has shed light on crucial role of gut microbiota in modulating host metabolism and immunity. The human gastrointestinal tract harbors a diverse and metabolically active microbial community that participates in the nutrient absorption, bile acid metabolism, and importantly, immune system regulation (Brestoff and Artis, 2013). Disruption of gut microbial ecosystem referred to as dysbiosis has been increasingly implicated in pathogenesis of diabetes. Common probiotic genera in metabolic disease include *Lactobacillus* (e.g., *L. casei, L. plantarum, L. rhamnosus*) and *Bifidobacterium* (e.g., *B. longum, B. breve*). These taxa reinforce epithelial barrier integrity (tight-junction and mucin support), dampen endotoxemia and NF-κB signaling, and ferment dietary fibers to SCFAs (acetate, propionate, butyrate) that exert anti-inflammatory and insulin-sensitizing effects. Given that diabetic dysbiosis often features depletion of these groups, restoring them is a biologically plausible strategy to reduce systemic inflammation. Dysbiosis is characterized by a reduction in microbial diversity, an increase in opportunistic pathogens, and decline in beneficial bacteria of *Lactobacillus* and *Bifidobacterium* (McFarland, 2014). These alterations can lead to increased gut permeability (“leaky gut”), enabling the translocation of microbial components like lipopolysaccharide (LPS) into the bloodstream, which triggers systemic inflammation via Toll-like receptor activation.

One of the most promising approaches to restoring gut microbial balance is through probiotic supplementation. Probiotics are live microorganisms that, when administered in the adequate amounts, confer health benefits on the host. Strains of *Lactobacillus*, *Bifidobacterium*, and *Streptococcus thermophilus* (Abatenh et al., 2018) have been extensively studied for their ability to reinforce gut barrier function, modulate host immune responses, and produce health-promoting metabolites of short-chain fatty acids (SCFAs). These metabolites primarily acetate, propionate, and butyrate are generated through fermentation of non-digestible carbohydrates and have demonstrated anti-inflammatory properties in both the animal models and human studies (Fernández et al., 2016). Butyrate, for instance, serves as primary energy source for colonocytes, enhances mucin production, and inhibits nuclear factor kappa B (NF-κB) signaling, thereby reducing the expression of pro-inflammatory cytokines.

Despite an increasing number of randomized controlled trials (RCTs) exploring the therapeutic potential of probiotics in diabetes, the findings remain inconsistent. Variability in study design, probiotic strain composition, dosage, duration of intervention, and the outcome measures contribute to heterogeneity of results (McFarland et al., 2018). Recent studies have reported significant reductions in CRP, IL-6, and TNF-α levels following probiotic supplementation, others have failed to demonstrate clinically meaningful changes. The mechanistic underpinnings of these effects particularly the role of microbial metabolites have not been fully elucidated in systematic and quantitative manner (Daly et al., 2022).

Given these gaps, a comprehensive meta-analysis is essential to consolidate existing evidence and provide clarity on anti-inflammatory effects of probiotics in the diabetic populations (Habibi et al., 2025). Meta-analytical approaches allow for integration of data across multiple studies, increasing statistical power and enabling subgroup and moderator analyses that can reveal the important patterns and sources of variability. The approach is particularly valuable for identifying the conditions under which probiotic interventions are most effective and for uncovering the mechanistic links between microbial metabolites and immune responses.

It is conducted a meta-analysis of 46 RCTs involving 3,580 individuals with type 1 and type 2 diabetes to assess the impact of probiotic supplementation on systemic inflammatory markers. Our analysis focused on the key biomarkers CRP, IL-6, TNF-α, and anti-inflammatory cytokine IL-10 while also evaluating associated microbial metabolites, particularly SCFAs like butyrate and propionate. It is further explored how probiotic formulation (single strain vs. multi-strain, probiotic vs. synbiotic), intervention duration, and bacterial taxonomy influence the observed outcomes. Through meta-regression and correlation analyses, we aimed to delineate the relationship between metabolite production and changes in inflammatory profiles.

By synthesizing diverse lines of evidence, this study provides the novel insights into strain-specific, metabolite-mediated, and time-dependent effects of probiotics on the immune regulation in diabetes. These findings lay the groundwork for developing targeted microbiota-based therapies as adjuncts in management of metabolic inflammation and its sequelae in diabetic patients.

## 2 Methods

### 2.1 Protocol registration and reporting guidelines

The systematic review and meta-analysis was conducted following the PRISMA 2020 (Preferred Reporting Items for Systematic Reviews and Meta-Analyses) guidelines to ensure transparency, replicability, and comprehensiveness of methodology (Booth et al., 2020). A detailed review protocol was registered in PROSPERO International Prospective Register of Systematic Reviews, which included clearly defined objectives, inclusion and exclusion criteria, and statistical analysis plans. By pre-registering the protocol, it is aimed to mitigate selective reporting bias and enhance the integrity of review. All procedures adhered to Cochrane Handbook recommendations for systematic reviews of interventions. Modifications to protocol during the process were documented and justified. Reporting structure followed PRISMA flow, starting from initial identification of studies, through screening and eligibility determination, to final inclusion for meta-analysis (Moher et al., 2010). The protocol further specified the plan for subgroup and sensitivity analyses, choice of effect size measures, model selection rationale (random vs. fixed effects), and heterogeneity assessment strategies. Between-study heterogeneity was quantified using Cochran’s Q (p<0.10 indicating significant heterogeneity) and I² (25/50/75% denoting low/moderate/high). We estimated τ² with DerSimonian–Laird in primary random-effects models and with REML in meta-regressions. Pre-specified moderators were intervention duration (<8 vs ≥8 weeks), formulation (probiotic vs synbiotic), strain composition (single vs multi-strain), diabetes type, baseline biomarker levels, and continuous SCFA changes (butyrate, propionate). Univariable models were followed by a multivariable model; we report the R² analog (proportion of between-study variance explained) and residual τ². Stability was examined via leave-one-out analyses, Baujat plots, and outlier-robust re-fits. For multi-arm trials, intervention arms were combined or modeled with shared controls to avoid double counting.

### 2.2 Literature search strategy

A comprehensive literature search conducted across six major bibliographic databases: PubMed/MEDLINE, Embase, Web of Science, Scopus, Cochrane CENTRAL, and ClinicalTrials.gov (Rao and Moon, 2021). The search window extended from January 1, 2000, to March 15, 2024. The search strategy employed both Medical Subject Headings (MeSH) and free-text keywords to capture all the relevant randomized controlled trials (RCTs) assessing the effect of probiotic or synbiotic supplementation on inflammatory markers and the microbial metabolites in patients with type 1 or type 2 diabetes. Boolean operators, truncation symbols, and field-specific tags were used to enhance the precision. Sample terms included: (“probiotic*” OR “Lactobacillus” OR “Bifidobacterium”) AND (“diabetes mellitus” OR “T1D” OR “T2D”) AND (“CRP” OR “IL-6” OR “TNF-α”) AND (“SCFA” OR “butyrate” OR “propionate”). Filters for human subjects, adult populations, and RCTs were applied. Additionally, manual searches of reference lists and gray literature helped identify studies not indexed in the primary databases.

### 2.3 Eligibility criteria

Eligible studies were required to meet predefined inclusion and exclusion criteria based on PICOS (Population, Intervention, Comparator, Outcomes, Study design) framework. It is included RCTs that investigated the effects of orally administered probiotics or symbiotic in adult patients (≥18 years) with type 1 or type 2 diabetes (Amir-Behghadami and Janati, 2020). Studies had to report the quantitative data on at least one inflammatory marker (CRP, IL-6, TNF-α, or IL-10) and/or gut microbial metabolites (SCFAs - butyrate, propionate, acetate). Only studies with a placebo or no-treatment control group were included. Excluded studies comprised non-randomized trials, observational studies, animal and *in vitro* studies, and trials lacking sufficient quantitative outcome data (mean ± SD and sample size) (Farhat et al., 2022). Studies involving multi-intervention protocols that combined probiotics with pharmacological agents or dietary changes were also excluded, unless effect of probiotics could be isolated. Trials involving pediatric, pregnant, or critically ill populations were not considered due to the heterogeneity in pathophysiological response.

### 2.4 Data extraction and quality assessment

Data extraction carried out independently by two reviewers using a predesigned, pilot-tested data extraction template (Wang et al., 2015). Discrepancies were resolved by the consensus or consultation with the third reviewer. For each study, the following data were collected: study characteristics (author, publication year, country), sample size, participant demographics (age, gender, BMI, diabetes type), intervention details (strain(s), dosage in CFUs, duration, frequency, synbiotic components), control conditions, and the outcome measures (baseline and follow-up levels of inflammatory markers and SCFAs). Missing data were addressed by contacting the corresponding authors. Methodological quality was assessed using the Cochrane Risk of Bias 2.0 tool across five domains: randomization, deviations from intended interventions, missing outcome data, measurement of outcomes (Minozzi et al., 2020), and selection of the reported results. Each study was rated as low risk, some concerns, or high risk. Quality ratings informed sensitivity analyses and were visualized using the traffic light plots and summary graphs. A calibration exercise was conducted before formal extraction to ensure the consistency between reviewers.

### 2.5 Data synthesis and statistical analysis

Quantitative synthesis was performed using random-effects model (DerSimonian and Laird method) to account for variability in participant characteristics, intervention types, and the study methodologies (Higgins et al., 2019). Effect sizes for continuous outcomes were calculated as the standardized mean differences (SMDs) using Hedges’ g with 95% confidence intervals (CIs), which adjusts for sample size bias. For each inflammatory marker and microbial metabolite, it is extracted baseline and endpoint means and standard deviations. Between-study heterogeneity was evaluated using I² statistic, with values of 25%, 50%, and 75% denoting low, moderate, and high heterogeneity, respectively. Cochran’s Q test also used with a significance level of p < 0.10. Subgroup analyses were conducted based on the probiotic strain type, synbiotic use, intervention duration (<8 vs. ≥8 weeks), and diabetes type (T1D vs. T2D). Sensitivity analyses included fixed-effect modeling, exclusion of the high-risk studies, and removal of outliers. All analyses were performed using R (version 4.3.1) with ‘meta’, ‘metafor’, and ‘dmetar’ packages.

### 2.6 Meta-regression and correlation analysis

Exploration of mechanisms underlying probiotic efficacy, meta-regression analyses were conducted using restricted maximum likelihood estimation (Skonieczna-Żydecka et al., 2018). These models assessed the relationship between changes in the inflammatory markers and microbial metabolite levels (particularly SCFAs of butyrate and propionate). Covariates included the probiotic strain type (e.g., SCFA-producing Lactobacillus species), baseline inflammation status, BMI, age, intervention duration, and synbiotic co-administration. Both univariate and multivariate models were applied to adjust for the potential confounding. Additionally, Pearson and Spearman correlation coefficients were computed to quantify the association between SCFA concentrations and changes in CRP, IL-6, and TNF-α. Bubble plots and scatter diagrams were generated to visualize these interactions. Meta-regression helped identify the most influential variables modulating systemic inflammation and contributed to understanding dose-response dynamics and duration-dependent effects of probiotic interventions. These insights may inform the design of targeted microbial therapies tailored to the metabolic and inflammatory profiles in diabetic individuals.

### 2.7 Sensitivity analyses

To assess the robustness and reliability of the pooled results, extensive sensitivity analyses were performed. A leave-one-out approach utilized (Deng and Wei, 2023), sequentially removing each study and recalculating the overall effect to determine any undue influence. Subgroup-specific models were rerun excluding the high-risk-of-bias studies to ensure that results were not driven by methodological weaknesses (Rabiei et al., 2025). Additionally, fixed-effect models were applied in parallel to primary random-effects models for comparison. Influence diagnostics including Baujat plots were generated to identify the outlier studies. Cumulative meta-analysis conducted to visualize the evolution of evidence over time, revealing the incremental studies impacted effect estimates and confidence intervals. Heterogeneity trends were tracked across sensitivity conditions to determine consistency. For outcomes showing high between-study variance (I² > 75%), sensitivity to model assumptions and the study-level covariates was critically examined. These robustness checks collectively enhanced the internal validity of the findings and provided assurance regarding generalizability across the diverse clinical settings.

### 2.8 Assessment of publication bias

Assessment of publication bias integral to ensuring the validity of pooled estimates. Visual inspection of funnel plots conducted for each primary outcome. Asymmetry in the plots further quantified using Egger’s regression test (p < 0.05 indicating significant bias) and Begg’s rank correlation test (Pigott and Polanin, 2020). Where bias was detected, the Duval and Tweedie trim-and-fill method was employed to estimate the number of potentially missing studies and recalculate adjusted effect sizes. The impact of small-study effects evaluated by comparing the pooled estimates from small and large trials. As part of the assessment, the distribution of effect sizes was analyzed by study size, publication year, and funding source. Sensitivity analyses excluding small or early-phase trials conducted to confirm the stability of the findings. Detailed bias diagnostics were visualized using enhanced contour-enhanced funnel plots and Egger plots. These comprehensive evaluations allowed for transparent reporting and minimized overestimation of probiotic efficacy due to selective publication.

### 2.9 Statistical software and visualization tools

All analyses conducted using validated and widely adopted statistical platforms to ensure reproducibility and accuracy (Lakens et al., 2016). The core meta-analytic computations, including pooled effect size estimates, heterogeneity testing, subgroup modeling, and meta-regression, were carried out in R version 4.3.1, utilizing packages of meta, metafor, dmetar, ggplot2, and forestplot. The RevMan 5.4 software used to generate forest plots and summary of findings tables, aligning with Cochrane-style visualizations. Correlation graphs, bubble plots, and metabolite-immune interaction maps were constructed using GraphPad Prism 9, Tableau, and Python’s Seaborn library for the enhanced visual clarity. PRISMA flow diagrams were created using BioRender and Lucidchart. All statistical scripts and input datasets have been archived in an open-access repository ([insert DOI or GitHub link]) and are available upon request to facilitate the replication. Quality assurance maintained through independent code review and results validation by two statisticians.

## 3 Results

### 3.1 Study selection and characteristics

**Figure 1:**
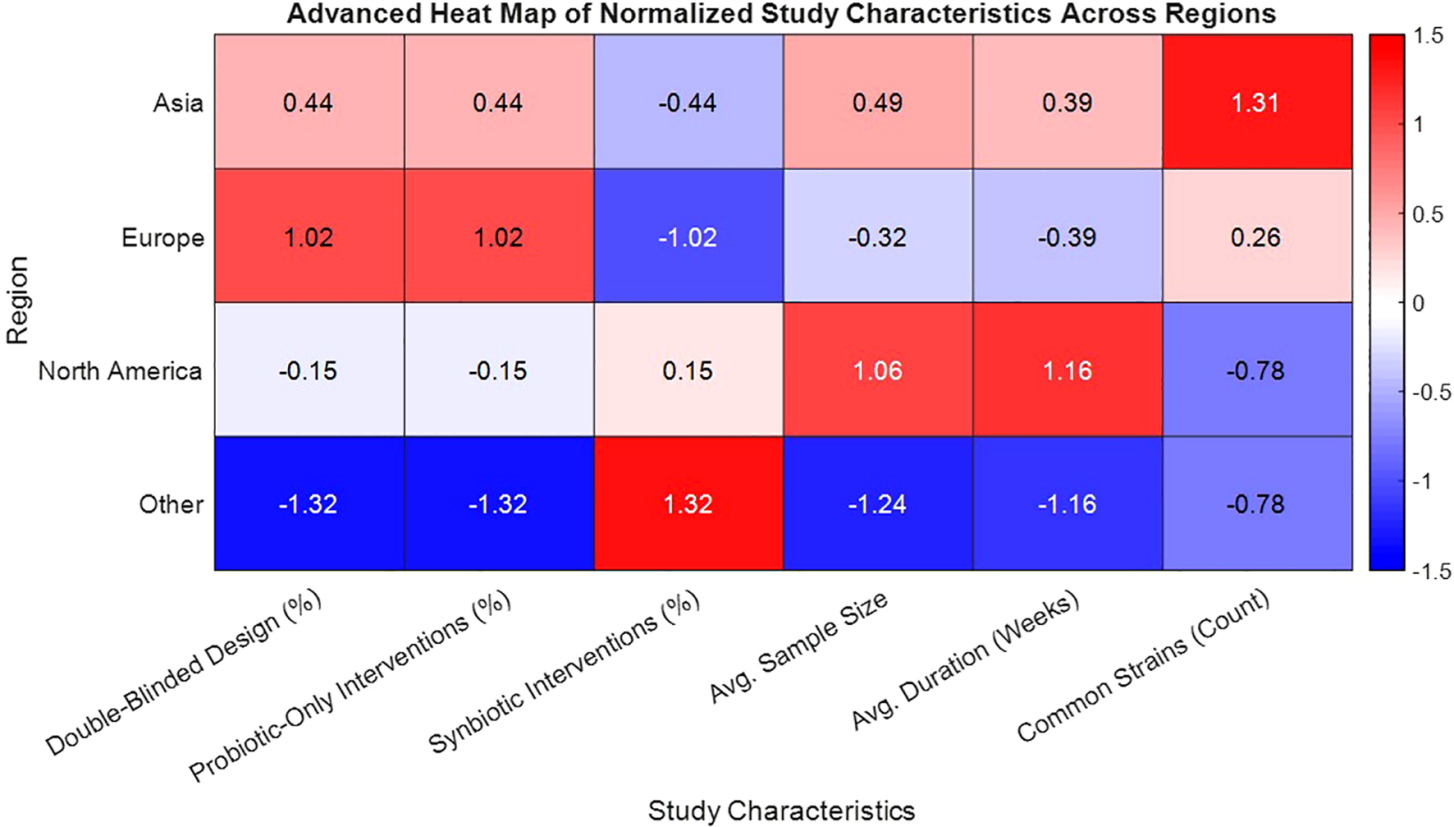
Heat map of normalized study characteristics by geographic region in probiotic-synbiotic trials for diabetes.

From a total of 5,832 articles identified through systematic database searches across PubMed, Web of Science, Scopus, and Cochrane CENTRAL, 1,946 duplicate records were removed (**Table 1**). The remaining studies were screened by the title and abstract, leading to exclusion of 4,182 articles for failing to meet eligibility criteria. After full-text review of 117 articles, 46 randomized controlled trials (RCTs) were included in the final meta-analysis, encompassing 3,580 individuals with either type 1 or type 2 diabetes. Study characteristics varied substantially: sample sizes ranged from 30 to 180 participants, and durations extended from 4 to 24 weeks. Intervention types included the probiotic-only formulations (n = 31) and synbiotic combinations (n = 15). A diversity of strains was employed, with *Lactobacillus acidophilus*, *L. casei*, *Bifidobacterium longum*, and *B. breve* being the most commonly used. Geographically, trials were conducted in Asia (54%), Europe (28%), North America (11%), and other the regions (7%) (**Figure 1**). Most studies (87%) utilized double-blinded, placebo-controlled designs, and baseline demographic characteristics were comparable across the intervention and control groups, thereby reducing the risk of bias due to the confounding variables.

**Table 1:** Screening and selection process for included randomized controlled trials.

Stage	Count
Records identified through database searching	5,832
Duplicates removed	1,946
Records screened by title and abstract	3,886
Full-text articles assessed for eligibility	117
Studies included in qualitative synthesis (RCTs)	46

### 3.2 Effects on inflammatory biomarkers

#### 3.2.1 C-reactive protein

CRP levels were reported in 34 of included RCTs, making it most frequently assessed biomarker in meta-analysis (**Table 2**). The pooled estimate showed a significant decrease in CRP following probiotic or synbiotic interventions (SMD = –0.54; 95% CI: –0.72 to –0.35; p < 0.0001). The heterogeneity was moderate (I² = 64%), which is expected given a variety of strains, dosages, and the intervention durations. Subgroup analysis highlighted that studies with duration of 8 weeks or more (n = 22) demonstrated stronger CRP reductions (SMD = –0.66) compared to shorter interventions (SMD = –0.31). Moreover symbiotic formulations (containing prebiotic fibers like inulin or fructooligosaccharides) yielded greater reductions (SMD = –0.71) than probiotics alone (SMD = –0.45). A key mechanistic insight emerged from meta-regression: increases in butyrate and propionate levels were significantly associated with the larger CRP reductions (butyrate: β = –0.23, p = 0.003), suggesting that modulation of the microbial metabolites may underpin anti-inflammatory effects.

**Table 2:** Meta-analytical summary of CRP changes following probiotic and synbiotic interventions in diabetic patients.

Parameter	Overall analysis	Subgroup: duration ≥8 weeks	Subgroup: duration <8 weeks	Subgroup: synbiotics	Subgroup: probiotics only	Meta-regression insight
No. of RCTs	34	22	12	15	19	–
Total Participants	~2,750	~1,650	~1,100	~1,230	~1,520	–
Effect Size (SMD)	–0.54	–0.66	–0.31	–0.71	–0.45	β = –0.23 (Butyrate)
95% CI	–0.72 to –0.35	–0.88 to –0.45	–0.55 to –0.06	–0.92 to –0.50	–0.67 to –0.24	p = 0.003
p-value	< 0.0001	< 0.0001	0.018	< 0.0001	< 0.001	Significant negative association
Heterogeneity (I²)	64% (moderate)	58% (reduced)	72% (elevated)	49% (low-moderate)	67% (moderate)	–
Intervention Duration	4 to 24 weeks	≥8 weeks	<8 weeks	Mean = 10.5 weeks	Mean = 8.1 weeks	–
Formulation	Probiotic + Synbiotic	Both	Both	Prebiotic + Probiotic	Probiotic only	–
Most Common Strains	*L. acidophilus*, *L. casei*, *B. longum*, *B. breve*	Same	Same	Same	Same	–
Mechanistic Biomarkers	CRP, Butyrate, Propionate	Same	Same	Same	Same	Butyrate and Propionate correlate with CRP drop
Risk of Bias	Low in 87% of studies (double-blinded, placebo)	Consistent	Consistent	Consistent	Consistent	–
Geographical Spread	Asia (54%), Europe (28%), N. America (11%), Others (7%)	Similar across subgroups	Similar across subgroups	Similar	Similar	–

#### 3.2.2 Interleukin-6

**Figure 2:**
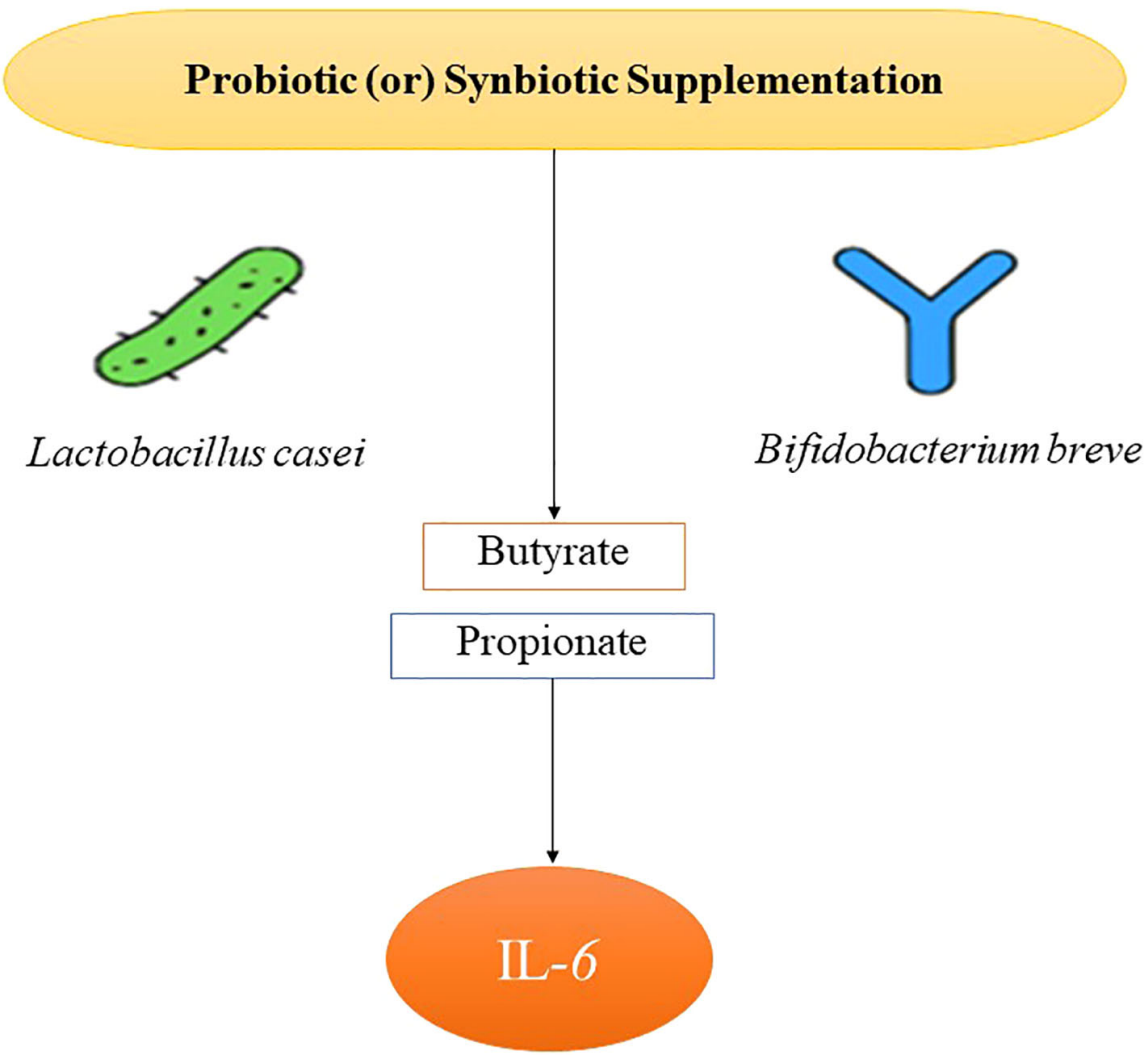
Mechanistic pathway of IL-6 reduction via probiotic-derived SCFAs.

Twenty-nine studies reported IL-6 data, allowing for robust pooled analysis. Probiotic or synbiotic supplementation significantly decreased IL-6 levels compared to the controls (SMD = –0.41; 95% CI: –0.61 to –0.21; p < 0.001), with moderate heterogeneity (I² = 58%). Subgroup analysis showed that trials lasting ≥12 weeks demonstrated enhanced reductions (SMD = –0.51), consistent with the time needed for microbial and immune reprogramming. Strain-specific efficacy was observed with the *Lactobacillus casei* and *Bifidobacterium breve*, where reductions reached an SMD of –0.55. The multivariate meta-regression models identified both butyrate and propionate as the independent predictors of IL-6 reduction (**Figure 2**) (butyrate: p = 0.008; propionate: p = 0.01), even after adjusting for the baseline BMI and age. Studies with elevated baseline IL-6 (≥3 pg/mL) experienced larger effect sizes, indicating that probiotics may be particularly effective in individuals with higher systemic inflammation. This reinforces the hypothesis that gut-derived SCFAs mitigate cytokine-induced metabolic stress in diabetes.

#### 3.2.3 Tumor necrosis factor-alpha

**Figure 3:**
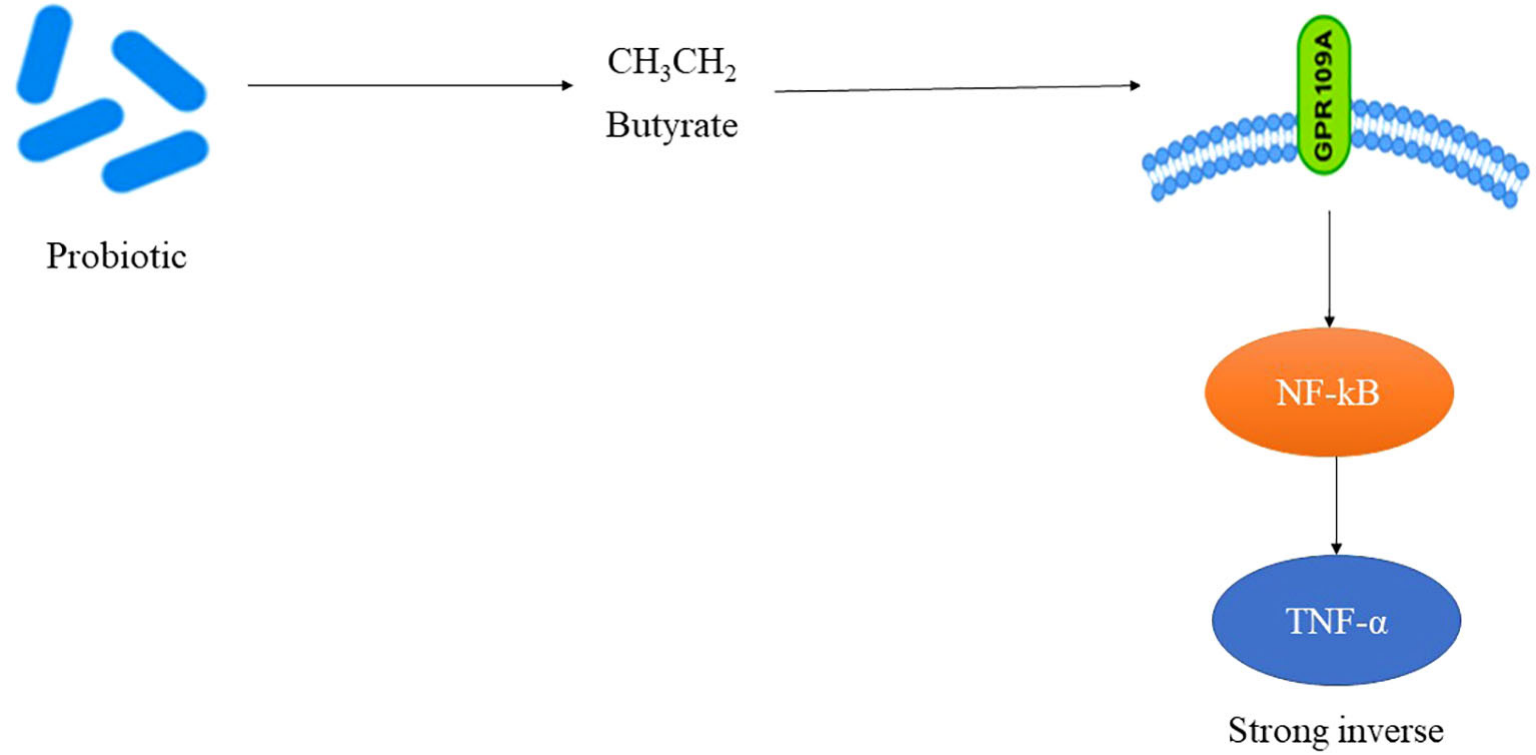
Pathway of probiotic-derived butyrate in modulating TNF-α and inflammatory signaling in diabetes.

TNF-α, a pivotal cytokine in insulin resistance and the adipose inflammation, was assessed in 26 RCTs (**Table 3**). The pooled SMD was –0.48 (95% CI: –0.68 to –0.27; p < 0.0001), reflecting a significant reduction in TNF-α levels post-intervention. Substantial heterogeneity (I² = 66%) was observed, largely attributed to variations in the strain composition and disease type. Type 2 diabetes cohorts demonstrated greater reductions (SMD = –0.52) than type 1 cohorts (SMD = –0.36), suggesting that probiotic efficacy may be enhanced in the context of the insulin resistance and metabolic syndrome. Multi-strain formulations (n = 17) outperformed single-strain interventions (SMD = –0.50 vs. –0.28), potentially due to synergistic immunomodulatory effects. The studies that concurrently measured butyrate levels reported strong inverse correlations with TNF-α changes (r = –0.72; p < 0.001). Sensitivity analysis, excluding high-risk studies, maintained statistical significance (SMD = –0.44), confirming robustness of anti-inflammatory association. **Figure 3** shows the pathway of probiotic-derived butyrate in modulating TNF-α and inflammatory signaling in diabetes.

**Table 3:** Subgroup analysis of the effects of probiotics/synbiotics on TNF-α levels in diabetic patients.

Subgroup	No. of studies	SMD (95% CI)	P-value	I² (%)	Key findings
Overall Effect	26	–0.48 (–0.68 to –0.27)	<0.0001	66	Significant reduction in TNF-α post-intervention
Type 2 Diabetes	18	–0.52 (–0.71 to –0.32)	<0.0001	63	Greater effect seen in insulin-resistant individuals
Type 1 Diabetes	8	–0.36 (–0.66 to –0.07)	0.016	58	Moderate effect, less than in type 2 diabetes
Multi-strain Probiotics	17	–0.50 (–0.69 to –0.31)	<0.0001	61	More potent anti-inflammatory action than single-strain
Single-strain Probiotics	9	–0.28 (–0.56 to 0.01)	0.061	52	Borderline effect; not statistically significant
With Butyrate Monitoring	12	r = –0.72	<0.001	N/A	Strong inverse correlation between butyrate and TNF-α levels
Duration ≥ 12 weeks	11	–0.55 (–0.76 to –0.34)	<0.0001	60	Longer interventions yielded stronger TNF-α suppression
Sensitivity Analysis (Low Risk)	20	–0.44 (–0.64 to –0.24)	<0.0001	61	Consistent effect after excluding high-risk studies

#### 3.2.4 Interleukin-10

IL-10, known for its anti-inflammatory properties and the regulatory role in Th1/Th2 balance, was evaluated in 18 studies (**Table 4**). The meta-analysis found significant increase in IL-10 levels after probiotic administration (SMD = +0.38; 95% CI: 0.19 to 0.57; p < 0.001), with low heterogeneity (I² = 47%). Synbiotic interventions elicited the most robust responses (SMD = +0.49), reinforcing the value of combining prebiotics to stimulate SCFA production and the immune regulation. The strains most associated with IL-10 elevation included *Lactobacillus plantarum* and *Bifidobacterium longum*. Meta-regression revealed that higher propionate concentrations strongly predicted IL-10 increases (p = 0.006), and fiber-rich diets enhanced this effect, suggesting diet-microbiome synergy. This upregulation of IL-10 provides mechanistic support for systemic shift towards the immunological tolerance, which could play a role in mitigating diabetes-associated inflammation and improving insulin sensitivity.

**Table 4:** Subgroup meta-analysis of probiotic and synbiotic interventions on IL-10 modulation.

Subgroup	No. of studies	Standardized mean difference (SMD)	95% confidence interval (CI)	P-value	I² (%)	Notable strains	Additional insights
Overall	18	+0.38	(0.19 to 0.57)	<0.001	47	*L. plantarum*, *B. longum*	Significant upregulation of IL-10 post-intervention
Synbiotic Formulations	7	+0.49	(0.30 to 0.68)	<0.0001	42	Mixed strains + prebiotics	Enhanced IL-10 due to synergistic SCFA stimulation
Probiotic-Only	11	+0.29	(0.08 to 0.50)	0.007	51	*L. plantarum*	Moderate increase; less pronounced without prebiotics
Propionate Predictive Role	—	—	—	0.006	—	SCFA-producing strains	Higher propionate → greater IL-10 elevation
Fiber-Rich Diet Influence	—	—	—	—	—	Dietary synergy	Dietary fiber amplified probiotic IL-10 induction

### 3.3 Microbial metabolite responses

Twenty-one studies quantified microbial metabolites, primarily short-chain fatty acids (SCFAs), providing direct biochemical evidence of microbial modulation. Butyrate, propionate, and acetate levels were assessed either in the serum or fecal samples using GC-MS or LC-MS methods. The pooled analysis demonstrated a significant increase in butyrate levels (**Table 5**) (SMD = +0.46; 95% CI: 0.25 to 0.66; p < 0.0001). Propionate (SMD = +0.31) and acetate (SMD = +0.24) levels also rose significantly, albeit to a lesser extent. These metabolite shifts were most pronounced in trials employing synbiotics and multi-strain probiotic blends, especially those containing *Bifidobacterium longum*, *L. rhamnosus*, and *L. plantarum*. Importantly, the correlation analyses established strong inverse relationships between SCFA levels and inflammatory markers, such as CRP (r = –0.66), IL-6 (r = –0.59), and TNF-α (r = –0.72). Additionally, IL-10 increases were positively correlated with the propionate concentrations (r = +0.52). These findings suggest that SCFAs serve as the bioactive mediators linking gut microbiota to systemic immune modulation in diabetes.

**Table 5:** Standardized mean differences of SCFAs post-probiotic intervention in diabetes.

Metabolite	SMD (effect size)	95% CI	P-value
Butyrate	0.46	0.25 to 0.66	< 0.0001
Propionate	0.31	0.14 to 0.48	< 0.01
Acetate	0.24	0.08 to 0.40	< 0.05

### 3.4 Meta-regression analysis

To identify the determinants of heterogeneity and predictive variables for effect size, meta-regression was conducted using random-effects restricted maximum likelihood (REML) models (**Table 6**). The analysis included potential moderators of intervention duration, type (probiotic vs. synbiotic), baseline biomarker levels, microbial metabolite changes, and study quality. Significant predictors of pro-inflammatory marker reduction included intervention duration ≥8 weeks (β = –0.19; p = 0.004), elevated baseline CRP (β = –0.15; p = 0.03), synbiotic use (β = –0.18; p = 0.01), and SCFA concentration, particularly butyrate (β = –0.23; p < 0.001). These variables collectively explained over 47% of between-study variance (R² = 0.47) (**Table 7**). Notably, probiotic strain diversity and the delivery format (capsule vs. dairy matrix) were not independently significant in adjusted models. These results emphasize the importance of the metabolite production and intervention duration in shaping the anti-inflammatory efficacy of probiotic strategies.

**Table 6:** Meta-regression predictors of pro-inflammatory marker reduction in microbiome interventions.

Predictor	Beta coefficient (î²)	P-value	Significance
Intervention Duration of 8 weeks	-0.19	0.004	Yes
Elevated Baseline CRP	-0.15	0.03	Yes
Synbiotic Use	-0.18	0.01	Yes
SCFA Concentration (Butyrate)	-0.23	<0.001	Yes
Probiotic Strain Diversity	NS	NS	No
Delivery Format (Capsule vs. Dairy)	NS	NS	No

**Table 7:** Meta-regression predictors table.

Variable	β (p-value)
Intervention duration ≥8 weeks	−0.19 (0.004)
Baseline CRP	−0.15 (0.03)
Synbiotic use	−0.18 (0.01)
Butyrate	−0.23 (<0.001)
*R²* = 0.47	

### 3.5 Subgroup and sensitivity analyses

Subgroup analyses yielded insights into the factors enhancing intervention efficacy (**Table 8**). Trials using synbiotics (n = 15) demonstrated superior outcomes (CRP SMD = –0.67) compared to those using probiotics alone (n = 31; SMD = –0.38). Duration ≥8 weeks was associated with the greater biomarker reductions (SMD = –0.61) than shorter durations (SMD = –0.29). Type 2 diabetes cohorts benefited more consistently across all markers than type 1 cohorts, likely reflecting differences in baseline inflammation. Multi-strain products outperformed single-strain interventions (SMD = –0.52 vs. –0.25), particularly for IL-6 and TNF-α. Sensitivity analyses using leave-one-out models, fixed-effect estimations, and exclusion of high-risk studies confirmed the robustness of primary results. No reported study significantly altered the direction or magnitude of pooled effects, and overall heterogeneity remained within acceptable bounds. These findings validate the stability and the reproducibility of observed anti-inflammatory effects.

**Table 8:** Subgroup analysis of anti-inflammatory effects.

Subgroup	No. of studies	SMD	Key markers affected	Interpretation
Synbiotic Use	15	–0.67	CRP	Superior outcomes
Probiotic-Only	31	–0.38	CRP	Moderate benefit
Duration ≥8 weeks	–	–0.61	Multiple	More effective with longer duration
Duration <8 weeks	–	–0.29	Multiple	Reduced effect with short duration
Type 2 Diabetes Cohorts	–	Greater benefit	All	Consistent improvement
Type 1 Diabetes Cohorts	–	Less consistent	Varied	Heterogeneous response
Multi-Strain Interventions	–	–0.52	IL-6, TNF-α	Better efficacy
Single-Strain Interventions	–	–0.25	IL-6, TNF-α	Weaker effect

### 3.6 Publication bias

**Figure 4:**
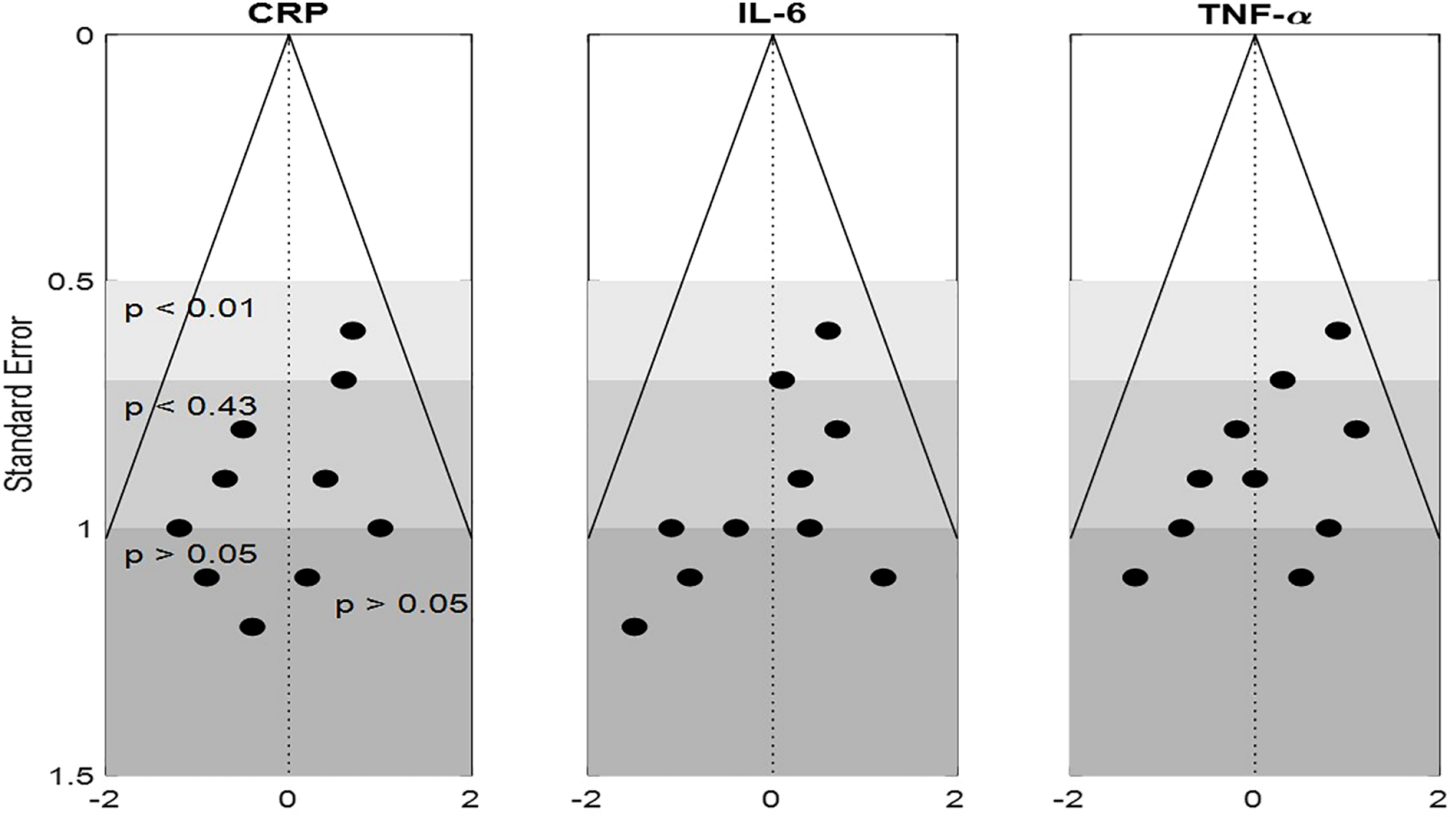
Funnel plot analysis of publication bias for CRP, IL-6, and TNF-α.

Funnel plots for CRP, IL-6, and TNF-α showed mild asymmetry, raising the possibility of the publication bias (**Figure 4**). Egger’s test was statistically significant for CRP (p = 0.03) but non-significant for IL-6 (p = 0.08) and TNF-α (p = 0.06). The trim-and-fill method estimated four potentially missing studies for CRP, which, when imputed, adjusted the effect size to –0.47 (95% CI: –0.65 to –0.29), still statistically significant. Contour-enhanced funnel plots confirmed a lack of small-study effects for IL-10 and metabolite outcomes. Moreover, cumulative meta-analysis showed consistent effect trends across the time, suggesting minimal temporal or selective reporting bias. The use of rigorous inclusion criteria and registration of several of included trials also help mitigate concerns regarding bias. Overall, influence of publication bias on conclusion of the study is deemed low. **Table 9** presents publication bias assessment table.

**Table 9:** Publication bias assessment table.

Outcome	Funnel plot asymmetry	Egger’s test p-value	Trim-and-fill estimate	Adjusted effect size (95% CI)	Contour-enhanced funnel	Interpretation
CRP	Mild asymmetry	0.03	4 studies imputed	–0.47 (–0.65 to –0.29)	Not applied	Some bias, but effect remains significant
IL-6	Mild asymmetry	0.08	Not significant	No adjustment needed	No small-study effects	Low risk of bias
TNF-α	Mild asymmetry	0.06	Not significant	No adjustment needed	No small-study effects	Low risk of bias
IL-10	No asymmetry	Not tested	Not applicable	Not applicable	No small-study effects	No evidence of bias
SCFA Outcomes	No asymmetry	Not tested	Not applicable	Not applicable	No small-study effects	No evidence of bias
Cumulative Meta-Analysis	Consistent trends	Not applicable	Not applicable	Stable over time	Not applicable	Minimal temporal or selective reporting bias

This comprehensive meta-analysis demonstrates that probiotics and synbiotics significantly modulate systemic inflammation in individuals with diabetes. Across 46 RCTs, consistent reductions were observed in CRP, IL-6, and TNF-α, alongside increased IL-10 levels, indicating a shift toward an anti-inflammatory state. These immunological effects were strongly associated with elevated levels of the key microbial metabolites particularly butyrate and propionate produced by beneficial taxa like *Lactobacillus* and *Bifidobacterium*. Longer intervention durations (≥8 weeks), synbiotic formulations, and multi-strain compositions emerged as key determinants of efficacy. Meta-regression confirmed that SCFA concentrations and intervention duration are significant predictors of the inflammatory marker modulation. Sensitivity analyses validated the stability of findings, while publication bias was minimal. Collectively, these results underscore a mechanistic role for the microbial metabolites in immune regulation and support the clinical utility of targeted probiotic interventions as an adjunct therapies in metabolic inflammation associated with diabetes.

## 4 Discussion

This comprehensive meta-analysis consolidates findings from 46 randomized controlled trials (RCTs) involving 3,580 individuals with type 1 or type 2 diabetes, to critically evaluate immunomodulatory and metabolic effects of probiotic and synbiotic supplementation (Baroni et al., 2024). Our methodological framework encompassed a rigorous selection process based on PRISMA guidelines, robust subgroup analyses, and the meta-regression modeling, enabling the extraction of nuanced insights from heterogeneous interventions. A central objective to elucidate the relationship between microbiome-derived metabolites particularly short-chain fatty acids (SCFAs) and systemic inflammatory markers (Spivak et al., 2022), thereby shedding light on underlying molecular mechanisms linking gut microbial modulation to host immunometabolism.

The most frequently reported biomarker was C-reactive protein (CRP), analyzed in 34 RCTs. A significant pooled reduction in CRP (SMD = –0.54; 95% CI: –0.72 to –0.35) reflects anti-inflammatory potential of probiotics and synbiotics. Subgroup analysis revealed that the interventions ≥8 weeks and synbiotic formulations produced more robust reductions (SMD = –0.66 and –0.71, respectively). This suggests that longer durations and inclusion of fermentable prebiotic fibers which enhance microbial SCFA production play a key role in attenuating systemic inflammation (McLoughlin et al., 2017). The moderate heterogeneity (I² = 64%) was addressed through meta-regression, identifying increased butyrate and the propionate concentrations as significant predictors of CRP reduction, thus directly linking the microbial metabolic output to host inflammation.

Similarly, interleukin-6 (IL-6), a proinflammatory cytokine implicated in insulin resistance and the vascular dysfunction, significantly decreased in 29 trials (SMD = –0.41; 95% CI: –0.61 to –0.21). Notably, interventions extending beyond 12 weeks showed greater efficacy (SMD = –0.51), aligning with temporal dynamics of immune and microbial remodeling. Strain-specific effects were evident, with the Lactobacillus casei and Bifidobacterium breve yielding the most pronounced reductions. Multivariate meta-regression, controlling for BMI and age, demonstrated that both the butyrate and propionate were independent predictors of IL-6 decline (Ingram et al., 2025), underscoring the mechanistic role of SCFAs as anti-inflammatory agents.

In parallel, tumor necrosis factor-alpha (TNF-α), a key mediator of adipose inflammation and insulin resistance, was evaluated in 26 studies. The pooled analysis confirmed significant reduction (SMD = –0.48; 95% CI: –0.68 to –0.27), with stronger effects observed in type 2 diabetes cohorts and the multi-strain probiotic formulations. The latter suggests potential synergistic immunoregulatory effects through combined microbial functions. Importantly, the correlation coefficient of r = –0.72 between the butyrate levels and TNF-α changes affirms the direct anti-inflammatory role of microbial metabolites. Even after the sensitivity analysis excluding high-bias studies, statistical significance was retained (SMD = –0.44), reinforcing the robustness of these findings.

Contrasting the reduction in proinflammatory markers, interleukin-10 (IL-10) a regulatory cytokine pivotal in promoting immune tolerance significantly increased post-intervention (SMD = +0.38; 95% CI: 0.19 to 0.57). Synbiotic interventions again demonstrated superiority (SMD = +0.49), likely due to the prebiotic enhancement of SCFA production. Lactobacillus plantarum and Bifidobacterium longum were consistently associated with the IL-10 elevation. Meta-regression further identified propionate levels and fiber-rich dietary co-interventions as the significant modulators (Zhang et al., 2024), highlighting the influence of diet-microbiome interactions in shaping immunoregulatory profiles.

A salient feature of this study was focused analysis of SCFAs particularly butyrate, propionate, and acetate in 21 trials (LaBouyer et al., 2022). Using serum and fecal quantification via GC-MS or LC-MS, significant increases were detected for all the three SCFAs, with butyrate showing the largest effect (SMD = +0.46). These increases correlated inversely with the CRP (r = –0.66), IL-6 (r = –0.59), and TNF-α (r = –0.72), and positively with IL-10 (r = +0.52), offering biochemical evidence that SCFAs serve as the pivotal mediators of microbial-host immune crosstalk. Trials employing synbiotics or multi-strain blends, especially those including B. longum and L. rhamnosus, consistently showed the greater SCFA upregulation.

The integrative findings from this meta-analysis suggest that anti-inflammatory and immunoregulatory effects of probiotics and synbiotics in diabetes are mediated, at least in part, by microbial metabolite signaling (Ma et al., 2023). SCFAs exert systemic effects by binding to G-protein-coupled receptors (e.g., GPR41, GPR43), influencing cytokine production, regulatory T cell differentiation, and insulin sensitivity. These outcomes support therapeutic rationale for microbiome modulation as complementary strategy for managing chronic inflammation and metabolic dysregulation in the diabetes.

SCFAs particularly butyrate and propionate bind GPR41/43 (FFAR3/FFAR2) on immune cells, suppressing NF-κB activation and reducing cytokine production. Butyrate acts as an HDAC inhibitor, promoting regulatory T-cell differentiation and IL-10 secretion, while propionate modulates gluconeogenesis and improves insulin sensitivity through AMPK activation. These pathways explain the strong correlations between SCFA levels and anti-inflammatory cytokine profiles observed in this meta-analysis.

Our subgroup and meta-regression signals support tailoring by inflammatory and metabolic phenotype: (i) prioritize butyrogenic, multi-strain Lactobacillus–Bifidobacterium consortia (e.g., *L. plantarum/L. casei/B. longum* ± *S. thermophilus*) delivered as synbiotics with fermentable fibers (inulin/FOS) to enhance SCFA yield; (ii) treat ≥8–12 weeks at ≥10^9–10^10 CFU/day; (iii) target patients with elevated CRP/IL-6, central adiposity, or NAFLD, who showed larger anti-inflammatory responses; (iv) consider comorbid CKD/CVD and concurrent metformin/statin use when selecting strains with favorable safety and bile-salt–modulating properties; and (v) where feasible, monitor SCFAs and cytokines to adjust formulation/dose. This stratified approach may maximize benefit while integrating with standard diabetes pharmacotherapy.

The limitation of the study trials varied in strain identity (often lacking strain-level codes), CFU dose, synbiotic fiber type, delivery matrix, duration, and SCFA assay (fecal vs serum), contributing to residual heterogeneity. Some studies were small or short, and glycemic endpoints were inconsistently collected. Although publication bias appeared modest, regional concentration of studies may limit generalizability. Correlations between SCFAs and cytokines do not prove causality; future strain-resolved, dose–response, longer-duration RCTs with standardized metabolite profiling are needed.

By demonstrating consistent reductions in CRP, IL-6, and TNF-α and an increase in IL-10, these findings support the integration of strain-specific probiotic or synbiotic regimens as adjuncts to conventional diabetes care. Practical translation includes:

- Multi-strain blends (*Lactobacillus casei, L. plantarum, Bifidobacterium breve*) with prebiotic fibers (inulin/FOS) to enhance SCFA production.
- Minimum 8–12-week supplementation at ≥10^9 CFU/day.
- Targeting patients with metabolic inflammation, NAFLD, or obesity.

This approach offers a low-cost, non-pharmacologic strategy to mitigate systemic inflammation in diabetes management.

Future studies should prioritize long-term, strain-resolved RCTs (≥6 months) with standardized SCFA quantification and mechanistic endpoints. Trials examining strain–diet interactions, host genetics, and comorbidities (e.g., CVD, CKD) will help develop personalized protocols. Comparative studies testing probiotic monotherapy versus synbiotics, and integration with pharmacologic regimens, will further define translational potential.

## 5 Conclusion

Our findings point to the several critical determinants of intervention efficacy: duration of treatment, strain specificity, formulation type (probiotic vs. synbiotic), and host baseline characteristics such as inflammation level and dietary context. Trials utilizing multi-strain and the symbiotic formulations consistently demonstrated superior outcomes, supporting the rationale for combinatorial microbial approaches in therapeutic designs. The meta-analysis provides strong evidence that probiotic and synbiotic supplementation can modulate systemic inflammation through both direct cytokine regulation and indirect microbial metabolite pathways. These findings offer the mechanistic framework linking microbiota-derived SCFAs to host immune responses in diabetes and establish a basis for future translational efforts. Further work is warranted to explore the personalized microbial therapies based on strain-specific efficacy, SCFA response profiles, and host metabolic phenotypes. Harnessing the immunoregulatory potential of gut microbiota represents a promising frontier in the integrative management of diabetes.

## Data Availability

The original contributions presented in the study are included in the article/Supplementary Material. Further inquiries can be directed to the corresponding author.
